# Diabetes mellitus promotes susceptibility to periodontitis—novel insight into the molecular mechanisms

**DOI:** 10.3389/fendo.2023.1192625

**Published:** 2023-08-16

**Authors:** Mingcan Zhao, Yuandong Xie, Wenjia Gao, Chunwang Li, Qiang Ye, Yi Li

**Affiliations:** ^1^ Department of Pediatric Dentistry, Hospital of Stomatology, Jilin University, Changchun, Jilin, China; ^2^ Jilin Provincial Key Laboratory of Tooth Development and Bone Remodeling, Hospital of Stomatology, Jilin University, Changchun, Jilin, China

**Keywords:** diabetes mellitus, inflammatory, host immune response, oxidative stress, microbiota, microRNA, epigenetic changes, periodontitis

## Abstract

Diabetes mellitus is a main risk factor for periodontitis, but until now, the underlying molecular mechanisms remain unclear. Diabetes can increase the pathogenicity of the periodontal microbiota and the inflammatory/host immune response of the periodontium. Hyperglycemia induces reactive oxygen species (ROS) production and enhances oxidative stress (OS), exacerbating periodontal tissue destruction. Furthermore, the alveolar bone resorption damage and the epigenetic changes in periodontal tissue induced by diabetes may also contribute to periodontitis. We will review the latest clinical data on the evidence of diabetes promoting the susceptibility of periodontitis from epidemiological, molecular mechanistic, and potential therapeutic targets and discuss the possible molecular mechanistic targets, focusing in particular on novel data on inflammatory/host immune response and OS. Understanding the intertwined pathogenesis of diabetes mellitus and periodontitis can explain the cross-interference between endocrine metabolic and inflammatory diseases better, provide a theoretical basis for new systemic holistic treatment, and promote interprofessional collaboration between endocrine physicians and dentists.

## 1 Introduction

Diabetes mellitus is a group of metabolic disorder diseases caused by absolute or relative insulin secretion insufficiency and (or) insulin utilization obstacles, with hyperglycemia as the main feature, causing a longer-term health aftermath, higher mortality risks, and reduced life expectancy (1). The 10th edition of the IDF Diabetes Atlas shows that there will be 537 million diabetes mellitus patients worldwide in 2021; more than one in 10 adults now have diabetes mellitus. It is estimated that by 2045, the number of diabetes patients will reach 783 million. The USA, India, China, and other countries with large populations contribute the most to the total number of diabetes mellitus patients (2). The high attack rate, disability rate, case fatality rate, and rapid growth prevalence of diabetes have brought major challenges to the healthcare system. Diabetes mellitus-related chronic complications can affect multiple organs throughout the body, including macro- and microvascular diseases, diabetes-related nephropathy, diabetes-related peripheral neuropathy, diabetes-related retinopathy, which may eventually lead to blindness, and diabetes-related delayed tissue healing (3). Since 1993, periodontitis has been described as another typical complication of diabetes mellitus (4). Furthermore, several reviews have indicated that diabetes mellitus patients are more likely to suffer from periodontal disease than those without diabetes (5–9).

Periodontitis is the result of the interaction between a repaired inflammatory/host immune response of the periodontium and a dysbiotic periodontal microbiota challenge (10) characterized by a chronic destructive and progressive inflammatory response, which is caused by gram-negative bacteria in a bacterial plaque within microbial dysbiosis (11, 12). It is a chronic multifactorial inflammation disease affecting the supportive tissues of the entire periodontium and may lead to loss of periodontal attachment, formation of periodontal pocket, resorption of alveolar bone, and eventually tooth loss if left untreated (13). Periodontitis affects about 45%–50% of adults, and the incidence of periodontitis among people over 65 years old rises to more than 60% (14–16). Severe periodontitis is the sixth most common human disease among 291 assessed global diseases (17), and it is estimated to affect an average of 11.2% (ranging from 5% in Oceania to 20.4% in Latin America) of the global adult population. In recent years, many epidemiological and experimental studies have demonstrated that periodontitis can affect systemic health through various molecular mechanisms and is independently related to most chronic systemic comprehensive diseases, such as diabetes mellitus (18). Therefore, severe periodontitis is a large social, economic, and healthcare burden, can increase global medical expenses and social inequality, and also has a significant influence on the human public health system (18).

The existing studies show a certain bidirectional association between periodontitis and diabetes; they increase the incidence of each other and are related to disease severity. Diabetes mellitus is a main risk factor for periodontitis (5–9, 19), and it has been showed that the incidence or the risk of progression of periodontitis in diabetes patients with inadequate control increased by 86% compared with nondiabetes patients or well-controlled diabetes patients (20). On the other hand, periodontitis patients exhibit poor glycemia control, a further risk of insulin resistance, and a higher prevalence of diabetes-related complications (10, 21–24). Many studies have attested that periodontal therapy can reduce the load of periodontal inflammation, which in turn has a positive effect on blood glycated hemoglobin levels (25–29). Therefore, it is necessary to screen patients with diabetes for periodontitis and vice versa (9).

However, since the two comorbidities of diabetes with periodontitis are independently associated and mediated by complex interactions among microbiome, inflammation, host immune response, oxidative stress, genetics, and other factors (30), the exact molecular mechanisms between them and the pathways between diabetes and periodontal tissue anatomy and physiological changes are still unclear. In the meantime, there is a lack of multi-disciplinary collaboration between endocrinologists and stomatologists when managing diabetes patients (31); in fact, only 30% of endocrine physicians report that they have ever referred their patients to a stomatosis clinic institution for oral health assessment (32). It is noteworthy that endocrine physicians need to be aware of periodontitis and its impact on glycemia control and the risk of insulin resistance (22), and patients with periodontitis should also be consulted about their increased risk of diabetes (33). The aim of this study is to review the latest clinical data on the evidence of diabetes promoting the susceptibility of periodontitis from epidemiological, molecular mechanistic, and potential therapeutic targets and discuss the possible molecular mechanistic factors such as microbiome factors, inflammation factors, host immune response factors, oxidative stress factors, periodontal tissue destruction factors, and epigenetic changes, focusing in particular on novel data on inflammatory/host immune response and oxidative stress. Understanding the intertwined pathogenesis of diabetes mellitus and periodontitis better may provide convenience to physicians and dentists for timely diagnosis and optimal management of these two diseases.

## 2 Diabetes is a main risk factor for periodontal disease

There are several types of diabetes mellitus, but type 1 diabetes mellitus (T1DM) and type 2 diabetes mellitus (T2DM) are considered the two main groups (2). T1DM is characterized by autoimmune damage to the pancreatic Langerhan islet β-cells, which prevents the pancreas from producing (enough) insulin. In contrast, T2DM, which makes up 90%–95% of all cases of diagnosed diabetes, is characterized by insulin resistance in conjunction with a relatively low level of insulin secretion.

In 1993, periodontitis was described as the sixth complication of diabetes mellitus (4). Since then, several studies have confirmed that diabetes mellitus is associated with a higher prevalence, incidence, severity, and progression of periodontitis in both T1DM and T2DM (5–8, 19–21, 34–36). Diabetes patients have a two to three times higher risk of developing periodontitis than the general population (34); the level of glycemic control is the key to determining risk (37); and the severity of periodontitis is rising to 10 times higher for smokers (38). In fact, diabetes mellitus is considered one of the two real risk factors of periodontal disease (39) and has been included in the “grading” component of the new classification of periodontal diseases by the European Federation of Periodontology (EFP) and the American Academy of Periodontology (AAP) (14, 39, 40).

A meta-analysis of 13 studies showed that diabetes increased the incidence or risk of progression of patients with periodontitis by 86% (20). There are also many studies that have confirmed that patients with poorly controlled diabetes have more serious clinical symptoms of periodontitis (such as exploratory bleeding (bleeding on probing (BOP)), plaque index (PI), clinical attachment loss (AL)) and periodontitis radiological parameters (such as marginal bone loss (MBL)) compared with those without diabetes or well-controlled diabetes (37, 38, 41–44). Diabetes mellitus can revise the expression of inflammatory-related cytokines such as tumor necrosis factor-α (TNF-α), interleukin-6 (IL-6), and interleukin-1β (IL-1β); increase the generation of reactive oxygen species (ROS) and its association with the oxidative stress index; disrupt the receptor activator of nuclear factor-kappa B (NF-κB) ligand (RANKL)/osteoprotegerin (OPG) axis; activate osteoclasts responsible for bone resorption; and destroy polymorphonuclear leucocyte activity, which in turn may accelerate the development of periodontitis (44).

In conclusion, periodontitis and diabetes affect each other, but the two-way relationship between them seems to exist independently of related risk factors. On the other hand, among the diagnosed patients with diabetes mellitus, periodontitis is associated with elevated glycosylated hemoglobin, poor glycemic control, and a higher incidence of diabetes-related complications (41–43).

### 2.1 Effect of T2DM on periodontitis

The majority of available studies on the bidirectional interactions between diabetes mellitus and periodontitis are based on T2DM because its global prevalence is higher and the onset age of T2DM is older. These factors combine to increase the danger of the intersection of T2DM and periodontitis. Most studies that explore the relationship between T2DM and periodontitis have clearly shown that the enhancement of periodontal damage index and the increment of periodontitis risk are associated with increased glycosylated hemoglobin levels. Although it is not proportional, the level of hyperglycemia is associated with an increased probability of periodontitis and later tooth loss (5, 7–9, 41–44).

A recent review summarized data on the two-way relationship between T2DM and periodontitis and found that T2DM increased the prevalence rate of periodontitis by 34% (RR = 1.34; 95% CI, 1.11–1.61), and T2DM patients had a 0.89-mm higher clinical attachment loss, 0.61-mm deeper periodontal pockets, 2.01 fewer teeth remaining, and 2.22 more teeth lost than T2DM-free participants. The two diseases promoted each other’s incidence rate and were related to the severity of the disease (8). Stöhr et al. (9) also conducted a systematic review and meta-analysis of the existing evidence on this topic with 15 observational studies, including 295,804 participants and more than 22,500 confirmed cases, and the result reported that diabetes was associated with an increased risk of onset and progression of adult periodontitis compared with people without diabetes mellitus (SRR = 1.24; 95% CI, 1.13–1.37), so diabetes patients should understand their higher risk of periodontal disease (9). Another cross-sectional clinical study also confirmed that T2DM patients had more serious tooth loss, and more than 95% of T2DM patients had some periodontal damage to varying degrees. These results could be used as baseline data to promote the collaborative and comprehensive management of diabetes (37). A study in an Italian population also assessed that a family history of T2DM, poor glycemic control, and C-reactive protein (CRP) levels could be the effective predictors of the onset and progression of severe periodontitis. For 1 unit of increase in serum HbAlc, the probability of severe periodontitis in patients increased by approximately 60% (44), and there was a higher risk in T2DM patients with higher glycosylated hemoglobin levels at baseline (22).

Therefore, the onset, duration, and severity of hyperglycemia play an important role in the development of chronic periodontitis (44). Hyperglycemia promotes the inflammatory/host response of periodontal tissues in a direct or indirect way via the advanced glycation end product (AGE) and its receptor (RAGE), which can alter the function of leukocytes and fibroblasts, promote the expression of proinflammatory cytokines, and increase the RANKL/OPG ratio, ultimately promoting osteoclast formation and alveolar bone absorption (7). According to the numbers of cross-sectional and longitudinal studies, it can be concluded that T2DM is a main risk factor for periodontitis and may be one of the pathogenesis of periodontitis (5).

### 2.2 Effect of T1DM on periodontitis

There have been a few clinical studies exploring the relationship between T1DM and periodontitis in the past, and there is significant heterogeneity between these studies in assessing clinical periodontal status. Because most studies on T1DM involve children or adolescents, the relationship between periodontitis and T1DM at the crossover level is not as clear as that with T2DM (45). Sanz et al. (22) concluded that there was no sufficient evidence to suggest a possible association between periodontitis and poor glycemic control in T1DM patients. Roy et al. (46) also proved that when comparing diabetes patients with healthy ones in terms of clinical attachment loss (CAL), the combined difference between T2DM patients (0.652; 95% CI, 0.465–0.840) was more than that within T1DM patients (0.691; 95% CI, 0.427–0.956).

On the other hand, numerous studies on diabetic children have shown that the incidences of periodontal disease in patients with T1DM are higher than in healthy ones. These findings suggest that there is a two-way relationship between T1DM and periodontal disease similar to T2DM (36, 47–50). A systematic review and meta-analysis demonstrated that patients with T1DM had higher PI, pocket depth (PD), BOP, and CAL compared with children or adolescents without diabetes, and all the results were statistically significant (49). Another systematic review of 37 related studies reported that compared to the systemically healthy controls, T1DM patients had poorer periodontal health, higher plaque scores, and greater periodontal destruction (47). Similarly, a recent systematic review also conducted a comprehensive analysis of 11 studies on this subject and confirmed that T1DM is a main risk factor related to the development of periodontitis. Compared with nondiabetic people, the proportion of T1DM patients affected by periodontitis has more than doubled, and periodontitis is more serious. There seems to be a great difference between subjects in different glycemic control states (36). Moreover, periodontal health in children and adolescents with T1DM deteriorates with poor diabetes control (48). Furthermore, compared to healthy children, the saliva secretion rate of T1DM children was significantly reduced (51), and the decrease in human beta-defensin (hBD)-3 concentration in saliva could partly explain why children with T1DM were more likely to suffer from periodontal disease (52).

Although the number of recent studies involving the relationship between periodontitis and T1DM has increased exponentially, there is still no general consensus on the causal cause–effect relationship between periodontitis and T1DM (45), and the connection between periodontitis and T1DM does not appear to be as strong as the link with T2DM (49). Many systematic reviews have failed to demonstrate the causal relationship between them. Thus, more longitudinal studies with larger sample sizes are needed to prove whether periodontitis is a cause of metabolic control worsening in T1DM children or a result of the onset and progression of T1DM.

## 3 The plausible pathogenic mechanisms of diabetes mellitus on periodontitis

The plausible pathogenic mechanisms of diabetes promoting the susceptibility of periodontitis are as follows: (1) microbiome factors; (2) enhanced inflammatory response via cytokines, adipokines, AGE/RAGE, and miRNAs; (3) host immune factors; (4) oxidative stress; (5) alveolar bone resorption damage; and (6) epigenetic changes.

### 3.1 Microbiome factors

“Microbial dysbiosis” is considered to be the pathogenesis of periodontitis, and the transformation of beneficial symbiotic microbial communities to pathogenic bacteria communities in subgingival plaque biofilms is the major initiation and causation of periodontitis (12). Different microbial communities adhere to the surfaces of the tooth root and are better protected from the effects of shear forces and ambient oxygen compared to the supragingival microbial communities (53). It is well known that periodontal microbial biofilms can cause host inflammation, leading to periodontal destruction and tooth loss (54).

Previous studies failed to reach a consensus on the presence of diabetes mellitus having an effect on the composition of the periodontal microbiota (55). Taylor et al. (56) concluded that there was no compelling evidence to demonstrate that diabetes mellitus (T1DM or T2DM) had an obvious impact on the periodontal microbiota; in addition, the level of glycemic control in diabetes patients did not affect the composition of subgingival bacteria biofilm (57).

With the rapid growth of next-generation sequencing technologies (NGST), a lot of studies in recent years indicated that diabetes mellitus could alter the composition and biodiversity of the subgingival microbiome. A case-control study showed that the composition of subgingival microbiota varies depending on the presence of periodontal disease and different glycemic statuses. In diabetes subjects, the numbers of Actinobacteria and Fusobacterium were significantly more abundant. Actinobacteria increased the probability of diabetes by 10%, and Fusobacterium increased the probability of diabetes by 14%, while the numbers of Proteobacteria were less abundant (58). Another research showed that the salivary microbiome of nondiabetic people and patients with diabetes histories had significant differences (59). There was a clear reduction in the diversity of biological and phylogenetic in the subgingival microbiota of diabetes and pre-diabetes patients in comparison with patients with normoglycemic subgingival microbiota (60). Furthermore, compared with healthy controls, the numbers of Pseudomonas genera, Haemophilus, Streptococcus, and Neisseria increased significantly in T2DM, as did the Firmicutes/Bacteroidetes ratio, but the numbers of Acinetobacteria reduced greatly (61). A recent study using the metagenomics shotgun sequencing found that the subgingival microbiome of T2DM patients with periodontitis had few red complex species genes, while the subgingival microbiome of healthy periodontal status individuals in patients with diabetes contained more orange complex species genes. This obvious difference led to an ultimate increase in the susceptibility of the oral microbiome of diabetes patients to periodontitis than nondiabetic individuals (62).

A lot of authors reported that diabetes mellitus reduced the richness and alpha diversity of the subgingival microbiome, and the reason was insufficient glycemic control (59–61, 63, 64). On the other hand, there were also some studies that showed that diabetes did not change the abundance and diversity of genera or phyla of subgingival microbiota in periodontitis patients but would strengthen the subgingival microbiota network connection in the periodontal tissue, thereby affecting the occurrence and development of periodontitis (65, 66). Mathur et al. (67) detected that compared with control groups, there was no big difference in the alpha diversity of T2DM patients’ salivary microbiota, but the relative abundance of 12 genera such as Atopobium, Parvimonas, and Aggregatibacter were partially reduced, and drug treatment for T2DM could partially restore the abundance of some salivary microbiota genera. Another study showed the same result: at the genus level, a decreased abundance of Neisseria, Porphyromonas, and Prevotella and an increased abundance of Selenomonas, Faecalibacterium, and Rothia were observed in the diabetic groups (68). However, even with the use of modern methodologies and omics methods, the understanding of the composition of subgingival bacterium under the condition of diabetes mellitus was still insufficient (69). More future studies of larger cohorts are needed to carry out the truth.

At present, the only consensus view is that diabetes mellitus can increase the pathogenicity of the periodontal microbiota. T2DM patients are more susceptible to periodontal pathogens and have a higher risk of periodontitis (62). The metabolism of galactose, mannose, and fructose, the mutual conversion of glucuronic acid and pentose, and the pathways of glycolysis may represent a potential microbial association between diabetes and periodontitis, and butyric acid is important in the interaction between them, affecting the bacterial secretion system and leading to a higher risk of periodontitis in T2DM patients (70). In periodontitis patients with or without diabetes mellitus, four microbial functional pathways associated with cell motility and signal transduction of virulence factors were enriched (62). Moreover, diabetes changes the pathogenicity of oral microbiota by increasing the production of interleukin-17 (IL-17) (71). In addition, treatment with the IL-17 antibody reduced the pathogenicity of subgingival microbiota, decreased neutrophil recruitment, and diminished proinflammatory factors IL-6 and RANKL, ultimately achieving a reduction of alveolar bone resorption (72).

Taken together, the inflammatory environment of the periodontium inducing selective pressure drives microbial dysbiosis and increases the pathogenicity of the periodontal microbiota, which will stimulate the host to produce greater inflammation, causing attachment loss and a deep periodontal pocket of periodontitis (55). Diabetes mellitus can alter the oral microbiota, and this effect is partially reversed only by giving antibodies of inflammatory mediators IL-17, RANKL, and IL-6, which indicates that diabetes mellitus, oral bacterial composition, and inflammation have a strong correlation (6).

### 3.2 Inflammation factors

Inflammation is the primary focus of previous studies that attempted to link diabetes mellitus and periodontitis because both disease conditions are strongly associated with overt inflammatory processes (7). The occurrence of TIDM is usually due to disorders in immune regulation, leading to the activation of the innate immune system and the expansion of autoreactive T and B lymphocytes produced by autoantibodies (73). The reason for the development of T2DM is the inability of pancreatic islet B cells to compensate for hyperglycemic levels caused by decreased incretin response and increased glucagon secretion (74). Functional impairment of natural killer and B cells, as well as changes in the proliferation of macrophages and T cells, lead to the progression of T2DM (75).

Although microbial dysbiosis has a direct impact on periodontal tissue, it is also important for microorganisms to mediate the amplified inflammatory response of susceptible hosts by forming strong adhesive biofilms on the surface of teeth during the progression of periodontitis (53). The accumulation of dental plaque leads to inflammation in the periodontal tissues, which then drives environmental changes and facilitates the growth of gram-negative bacteria. Further uncontrolled inflammation/immune responses largely promote tissue destruction (76). Inflammation is a protective response of periodontal support tissue to bacterial noxious stimuli, a process aimed at restoring balance by eliminating noxious stimuli such as pathogens. However, chronic dysfunctional inflammation caused by diabetes mellitus can lead to enlarged tissue destruction. Diabetes, smoking, chronic inflammatory diseases, and other risk factors worsen the condition of periodontitis and affect the therapeutic effect of periodontitis (13).

#### 3.2.1 Cytokines

Cytokines are substances secreted by immune cells or other cells that are recognized as inflammatory mediators and are related to the inflammatory reaction. Studies strongly show that diabetes and hyperglycemia directly induce a high inflammatory status in the infected periodontium, and the expression of proinflammatory cytokines is more significant in gingival tissue and crevice fluid of patients with diabetes periodontitis than in individuals with system health (77).

The most abundant data available concerns increased IL-1β, TNF-α, and IL-6. There is sufficient evidence to show that compared to patients with simple periodontitis, patients with combined diabetes and periodontitis have increased proinflammatory cytokines IL-6 and IL-1β, and there is a quantitative relationship between glycemic control and these cytokines (56, 57). On the other hand, a decreased secretion of anti-inflammatory cytokines such as transforming growth factor-beta (TGF-β), interleukin-4 (IL-4), and interleukin-10 (IL-10) may also lead to the aggravation of periodontal inflammation in diabetes patients (78). In conclusion, the hyperglycemia caused by diabetes can directly or indirectly reduce the expression level of anti-inflammatory cytokines such as IL-4, IL-10, and TGF-β1 and increase the expression level of proinflammatory cytokines such as IL-1β, IL-6, IL-8, IL-17, and TNF-α, all of which together enhance the periodontal inflammatory performance (79, 80).

In addition to cytokines, other proinflammatory mediators, such as metalloproteinases and chemokines, are also found to be associated with diabetes (4, 7, 71, 72, 77, 78, 81–84). Kim et al. (85) confirmed that the expression of matrix metalloproteinase-14 (MMP-14) was higher in the periodontal tissues of diabetes patients with poor glucose control. On the other hand, a reduction in proresolution mediators such as resolvins, protectins, maresins, and lipoxins may also contribute to enhanced periodontal inflammation (76), which involves three separate biosynthetic and potent mediator families. During the inflammatory process, the proinflammatory factors emit signals which can facilitate the synthesis of proresolution mediators such as resolvins, leading to the activation of the innate immune system and eliminating pathogens stimuli (86–88). Ozturk et al. (89) also confirmed that there were higher levels of proinflammatory peptide substance-P in the gingival crevicular fluid and serum of diabetic patients with poor glycemic control, which may aggravate the progression of periodontitis and cause greater periodontal destruction.

#### 3.2.2 Adipokines

There are multifarious types of adipokines, including adiponectin related to metabolic function, resistin and leptin related to endocrine function, complement factors related to immunity, angiotensinogen related to cardiovascular function, and so on (90). According to previous studies, some adipokines are involved in the occurrence and progression of periodontal disease and diabetes, but the existing evidence is insufficient. There are also many studies trying to research the expression of adipokines in the gingival crevicular fluid or serum and related mechanisms in periodontitis patients with or without diabetes, and the results are also unclear; further investigations are warranted.

Resistin is produced by various cells of the immune/inflammatory system (91, and seems to be the most widely researched adipokine linking periodontal disease and diabetes. As a proinflammatory factor, resistin promotes the release of interleukin-12 (IL-12) and TNF-α, induces the activation of NF-κB, and enhances the secretion of monocyte chemoattractant protein-1 (MCP-1) and IL-6 (92). Two studies showed comparable resistin levels in gingival crevicular fluid and serum between periodontitis with/without diabetics (93, 94). However, the other study showed significantly higher levels of resistin in serum and gingival crevicular fluid in periodontitis with T2DM than in the chronic periodontitis group (95). A recent study identified that the expression of resistin in chronic periodontitis was significantly higher compared to the periodontal health group, and the level of resistin was also higher in the periodontitis patients with T2DM. There was a strong positive correlation between resistin, glycosylated hemoglobin (HbA1c) level, and periodontal clinical parameters, with significant statistical differences (96). This is due to the chronic systemic inflammatory state caused by T2DM and the chronic local expression of proinflammatory resistin in the periodontal tissue (97). Similarly, high levels of resistin can affect the activity of osteoclasts, thereby affecting the absorption of alveolar bone. At the same time, T2DM changes the composition of periodontal bacterial pathogens in patients and activates the host immune response, resulting in the release of resistin by neutrophils (98). Therefore, resistin as a potential mediator between T2DM and chronic periodontitis deserves more attention.

Adiponectin is an anti-inflammatory, while leptin is a proinflammatory. T2DM can increase the levels of leptin and decrease the levels of adiponectin (99–101). Recent studies have found that compared to individuals with systemic health, the adiponectin level in periodontitis patients with diabetes is significantly lower and the leptin level is significantly higher (102, 103). Similarly, Kardeşler et al. (104) also showed that, compared to patients with periodontitis without T2DM, patients with T2DM and periodontitis had lower adiponectin levels and higher leptin levels. Adiponectin, leptin, visfatin, and chemerin play an important role in the occurrence, development, and diabetes-related complications of T2DM (105). The adiponectin receptor agonist AdipoRon (APR) can activate the endogenous adiponectin receptors to exert osteoanabolic effects and reduce osteoclast numbers and alveolar bone loss significantly. At the same time, APR can enhance stem cells’ osteogenic differentiation, reduce stromal cell-derived factor 1, which can promote stem cell migration, and ultimately promote the repair and regeneration of alveolar bone (106). Adiponectin and its agonists may be promising potential therapeutic targets for the clinical treatment of diabetes-related periodontitis, but the potential mechanism and clinical application need further research (107).

Other adipokines also play a role in the progress of diabetes-related periodontitis. A recent study found that progranulin in the saliva of diabetes patients with periodontitis was significantly reduced after nonsurgical periodontal treatment, considering that progranulin might be a target inflammatory marker for diabetes-related periodontitis (108). Another interventional comparative study showed that there were significant differences in the levels of omentin-1 in the saliva and serum of chronic periodontitis patients with or without T2DM (109). In addition, compared to periodontitis alone, periodontitis patients with T2DM have higher levels of plasminogen activator inhibitor-1 (PAI-1) in saliva and serum, suggesting a possible role of adipokines in the regulation of glycemic levels and periodontal inflammation (110).

#### 3.2.3 Advanced glycation end products and their receptor

AGEs are produced by the nonenzymatic oxidation of lipids, nucleic acids, and proteins caused by hyperglycemia in diabetes and accumulate in the periodontium, leading to sustained periodontal tissue destruction (111, 112). Periodontitis patients with diabetes have higher AGE accumulation levels compared to periodontitis patients without diabetes (112, 113). The accumulation of AGEs in the periodontal tissue of diabetes patients will increase the susceptibility to periodontitis and aggravate the existing periodontitis, but the exact mechanism is still unclear.

AGEs have been demonstrated to decrease osteoblast-related molecule mRNA expression, such as type 1 collagen, core-binding factor alpha 1 (Cbfa1), and osteocalcin, and increase inflammation-related molecules such as IL-1β and calcium-binding protein S100A8 (114). Another potential action of AGEs is to influence bone metabolism and inflammation through the regulation of sclerostin expression, which has an adverse effect on bone formation or bone metabolism in osteocyte cells and may aggravate periodontitis with diabetes (115). AGE2, a form of AGEs, can respectively upregulate the expression of Toll-like receptor 2 (TLR2) and RAGE in periodontal tissue and significantly stimulate the production of sclerotin and IL-6 in osteocytes. The presence of AGEs can also upregulate the levels of proinflammatory cytokines TNF-α and IL-6, which will further exacerbate inflammatory reactions (115).

In addition to its effects on the bone system, further studies showed that AGEs affected almost all types of cells in periodontal tissue directly or indirectly by binding to their receptors(RAGEs), including periodontal ligament stem cells (PDLSCs), gingival fibroblasts (GFs), gingival epithelial cells (GECs), and periodontal ligament cells (PDLCs) (80, 80). AGEs can inhibit the osteogenic differentiation of human periodontal ligament stem cells (hPDLSCs) by activating the classic Wnt/β-catenin pathway, reducing osteogenic potential and the regeneration and repair of alveolar bone (116). The accumulation of AGEs in human gingival fibroblasts (HGFs) can increase the protein and mRNA expressions of intercellular adhesion molecule-1 (ICAM-1), RAGE, and ROS through the NF-κB signaling pathways and MAPK pathways and aggravate periodontitis (117). It is also shown that AGEs stimulated the expression of RAGE and TLR2 by activating NF-κB, JNK-MAPK, and p38 signaling pathways in human gingival epithelial cells (hGECs), which could lead to increased activation of calprotectin in human gingival fibroblasts, including S100A9 and S100A8 (111). In human periodontal ligament cells (hPDLCs), the AGE/RAGE system can activate nucleotide-binding oligomerization domain-like receptors expression, such as NLRP1-inflammasome and NLRP3-inflammasome, promoting the inflammatory response through the NF-κB signaling pathway (118).

High concentrations of AGEs will cause the deterioration of diabetes periodontitis, and the siRNAs of RAGE can significantly reverse this result (111, 114, 115, 117, 119). The current research points to the beneficial effects of the administration of AGE inhibitors or blockers on periodontal tissue. The use of AGE inhibitors may improve periodontal inflammation, alleviate the damage of pathogenic bacteria to periodontal tissue, and ultimately improve the outcome of periodontal treatment. The huge potential of this method is obvious through the local application in periodontal tissue of new drugs such as vitamin C, which is an antioxidant and immunomodulator, to reduce the detrimental effect of diabetes mellitus on the periodontal tissue (120). Meanwhile, the natural antioxidant magnolol can alleviate periodontal inflammation for diabetes patients with periodontal disease by reducing the accumulation of AGEs, maybe through the nuclear factor erythroid 2-related factor 2 (Nrf2) signaling pathway (121).

In conclusion, chronic hyperglycemia increases the incidence/severity of periodontal diseases by inducing an exaggerated and prolonged inflammatory response driven by the accumulation of AGEs, which are considered to be the main pathogenesis of diabetes-related complications by inducing immune disorders and cell damage (122–124). AGEs can expand the release of proinflammatory mediators, enhance the immune inflammatory response of periodontal tissue, amplify oxidative stress by stimulating the production of ROS, and increase the susceptibility to periodontitis in diabetes patients (80). The administration of AGE inhibitors or blockers can partially reverse this result.

#### 3.2.4 MicroRNAs

MicroRNAs (miRNAs) are a group of noncoding small RNAs that function as master protein synthesis regulators that can act directly on target genes or indirectly on their transcription factors, which in turn silence the expression of the target gene (125). It has been reported that more than 60% of the translation of human coding genes is regulated by these highly conserved single-stranded miRNAs (126). It is reported that miRNA expression is tissue-specific, and the imbalance of miRNA expression is not only closely related to various diseases but also affects other normal protein expression (127). Therefore, they not only play an important role in the normal physiological process but also in the pathological development of various diseases. Much literature has shown that tissue-specific miRNAs can be used as diagnostic markers for metabolic diseases such as diabetes and inflammatory diseases such as periodontitis (128, 129), and most detectable miRNAs are concentrated in the exosomes (130). As promising disease markers, miRNAs are confirmed to coordinate immune inflammatory responses and have a strong upward potential in future research (131). Although very important, due to the relative novelty of the field of miRNAs, the role of miRNAs in the pathology and physiology of diseases needs to be further elucidated (132).

Some miRNAs may play an important role in the comorbid pathogenesis of diabetes mellitus and periodontitis. Recent studies demonstrated that, when compared to the control groups, there was significant overexpression of miR-223 in chronic periodontitis patients with or without T2DM (133, 134). MiR-223 can regulate innate immunity, leading to the recruitment and overactivation of neutrophils (135); therefore, miR-223 is related to the pathogenesis of chronic periodontitis and is highly likely to become a diagnostic marker for periodontal disease. MiR-223 is also helpful in the differential diagnosis of T2DM combined with periodontitis and periodontitis alone (133, 134, 136). In addition, miR-223 is correlated with proinflammatory cytokines TNF-α, anti-inflammatory cytokines TGF-β and IL-10, and periodontitis clinical parameters such as probing depth (PD) and clinical attachment loss (CAL) in varying degrees; it is also highly correlated with susceptibility to diabetes mellitus and glycemic control levels (134).

MiR-200b, which is induced by inflammatory cytokines, plays a key role in the early stages of inflammatory response, mainly participating in innate immunity and promoting the differentiation of various immune cells, such as neutrophils and macrophages (137). Moreover, the study found that in the chronic periodontitis group of T2DM, there was a significant difference in the serum level of miR-200b, which was significantly negatively correlated with IL-10 and positively correlated with TNF-α (133). Moreover, miR-203 may also play have a connecting effect on diabetes mellitus and periodontitis and have an additional characteristic as a specific marker for differential diagnosis of patients with or without diabetes, as well as distinguishing diabetes with periodontitis from diabetes without periodontitis (133, 138, 139). MiR-203 can also reduce inflammation in periodontal tissue by repressing TNF-α (139).

In addition to the above-mentioned, miR-146a, miR-146b, and miR-155 are also identified as promising biomarkers for diabetes mellitus and periodontal diseases (139–142). The gene targets of these miRNAs involve both innate and acquired immune responses, including natural killer cells and T lymphocytes (143), indicating its potential role in immune-related inflammation diseases such as periodontitis and metabolic diseases such as diabetes mellitus. MiR-155, miR-146a, and miR-146b can sense pathogen stimulation through pattern recognition receptors via the NF-κB signaling pathway (144). The combination of NF-κB-miRNA-146a and NF-κB-miR-155 regulatory loops can regulate the duration and intensity of inflammatory responses, so miR-146 and miR-155 can cross-talk and provide optimum NF-κB pathway activity and ultimately reduce inflammation (140, 145). This result was confirmed in other studies (139, 139, 141, 142). MiR-146a, miR-146b, miR-155, and miR-203 expression were higher in patients with periodontitis with or without diabetes; MiR-146a is the only reliable predictor of periodontitis in diabetes patients, and miRNA-155 is the most reliable predictor of periodontitis in nondiabetes patients (139). Since miR-155 is a main regulator of immunity/inflammatory response, the transcription level of miR-155 can discriminate between diabetes and nondiabetes conditions (146).

Another study showed that high glucose related to diabetes mellitus induced apoptosis of human periodontal ligament cells through a diminution of miR-221 and miR-222 expression and an increment of caspase-3, a validated target for these miRNAs, and had an effect similar to the inhibition of miR-221/miR-222 in control groups using antagomiRs (147). On the contrary, the expression levels of miR-214 in the periodontium of diabetes-associated periodontitis patients were increased (148). In addition, miR-126 can inhibit the inflammatory response of HGFs caused by hyperglycemia by directly targeting tumor factor receptor-associated factor 6 (TRAF6), so it may be a potential therapeutic target for periodontitis with diabetes (149).

Dysregulated expression of miRNA profiles mediates the inflammatory stress response by various inflammatory factors, so miRNAs play an important role in the occurrence and development of diabetes and periodontitis (150, 151). However, the explicit regulatory mechanism of miRNA in aggravating periodontitis in patients with diabetes mellitus is still unclear, and it is a direction for future research.

### 3.3 Host immune factors

#### 3.3.1 The pathogenesis of periodontitis

The interaction between periodontal microbiota and host immunity response is key to the pathogenesis and periodontium destruction of periodontitis. The size (big or small) of the immune response of host periodontal tissue in combating dysbiotic microbiota is very important because it determines the clinical manifestation of periodontitis and the response to periodontal treatment (12). Hyperactive immune responses can increase inflammation and degradation of periodontal tissue (152).

Periodontal pathogenic bacteria are recognized by pattern recognition receptors such as TLR2, TLR4, TLR7, TLR8, and TLR9 on the surface of periodontal cells, which can lead to the response of the proinflammatory cell, activation of neutrophils, and recruitment of macrophages/lymphocytes through p38 mitogen-activated protein kinase and NF-κB signaling pathways. Similarly, prostaglandin E2 (PGE2) released by macrophages and oxidative stress induced by neutrophils can cause more release of proinflammatory cytokines such as TNF‐α, IL‐1β, and IL‐6 and matrix metalloproteinases (MMPs) such as MMP-1, MMP-2, MMP-8, MMP-9, and MMP-13, which lead to local degradation of collagen fibers, loss of periodontal attachment, and resorption of alveolar bone, promote the progression of periodontitis, and enlarge inflammatory response and periodontal tissue (153–157).

The inflammatory response induced by the dysbiotic microbiota community includes interleukin-23 (IL-23) (158), which is produced by both macrophages and dendritic cells (DCs) in the connective tissue (159), which can increase the proportion of Th17 lymphocytes and change the ratio of T helper 17/regulatory T cells (Th17/Treg) to tilt the balance toward proinflammatory Th17 cells (160). IL-17, which is derived from Th17 cells, also plays an important role in periodontal destruction; it can cause collagen destruction mediated by neutrocytes, alveolar bone loss via RANK/RANKL pathways, and contribute to periodontitis (161–163).

Host genotype may be closely related to periodontal susceptibility (164). Moreover, both comorbidities such as diabetes and environmental factors such as smoking are also very important (165).

#### 3.3.2 Diabetes mellitus affects the immune response of periodontium

Diabetes mellitus can affect the innate and adaptive immune systems of the periodontium; both of them are thought to contribute to the progression of periodontitis.

Neutrophils are an important component of innate immunity, forming the first effective line of defense for the host against periodontal bacteria (166). Neutrophil homeostasis can maintain a balance between body protection and destruction, and any imbalance may cause periodontal tissue damage (167). In previous studies, it was found that the numbers of neutrophils were enhanced, but the functional activity of neutrophils (such as chemotaxis, phagocytosis, bactericidal function, and others) was impaired in periodontitis patients with T2DM (168), which would give rise to intracellular killing and respiratory burst (166) and increase the severity of periodontitis in diabetic patients. Dysfunctional neutrophils may cause periodontal tissue damage through the release of inflammatory mediators or tissue-degrading enzymes (169). In diabetic patients with poor glycemic control, neutrophils can also be activated in advance to increase the periodontal damage by increasing the activity of protein kinase C (PKC) (170). Poorly functioning neutrophils can enhance tissue damage, produce more superoxide and proinflammatory cytokines as well as chemokines, and increase the number of neutrophils in periodontal tissue (171, 172). In addition, in diabetes patients, neutrophils will overexpress a key enzyme, peptidylarginine deiminase 4 (PAD4), which is a nuclear citrullinating enzyme that is involved in the release of decondensed chromatin from neutrophils, inhibit the periodontal defense response and promote periodontal inflammation, and form the neutrophil extracellular traps (NETs), which, together with fibrin, are implicated in host defense against pathogens (173, 174). Furthermore, the levels of calprotectin (S100A8/A9), the major cytoplasmic protein in neutrophils, are significantly higher in periodontitis patients with T2DM than those in chronic periodontitis and healthy controls (175).

Macrophages are another cell type associated with immune response and are thought to contribute to periodontitis. Diabetes can increase the polarization of the M1 proinflammatory phenotype of macrophages, thus increasing the susceptibility and severity of periodontal disease, and the number of M2 anti-inflammatory phenotype macrophages is correspondingly reduced (6). Classic pathways activate M1 proinflammatory macrophages, which are involved in the production of inflammation, while M2 anti-inflammatory macrophages are involved in the downregulation of inflammation (176). The systemic metabolic changes caused by diabetes-induced hyperglycemia and insulin resistance can change the polarization and function of macrophages, enhance myelopoiesis, and lead to increased release of monocytes, which are the predecessors of macrophages (177). Li et al. (178) found that among subjects with diabetes periodontitis, macrophages stimulated by high glucose would lead to insufficient secretion of SIRT6 and induce disorder of SIRT6-miR-216/217 axis, accompanied by neutrophil apoptosis, impeded efferocytosis, and increased neutrophil extracellular traps, thus aggravating the inflammatory response of periodontitis. Neutrophils obliterate periodontal microorganisms by performing phagocytosis or releasing NETs, later starting apoptosis and immediate efferocytosis by triggering macrophages to prevent greater damage to periodontal tissue. However, chronic disunion in patients with diabetes usually leads to long-term inflammation and the persistence of neutrophils and macrophages, which will aggravate the periodontal inflammation (173–178).

Although neutrophils and macrophages play an important role in host immune response, it is reported that other myeloid cells, such as T cells, are also important in periodontitis with diabetes mellitus. The inflammatory environment by maintenance of innate immune cells, including neutrophils and macrophages, as well as related inflammatory mediators, can be affected by adaptive immunity (163). It is conducive to the differentiation of initial CD4+ T cells in Th17 inflammatory cells in diabetes patients, thus affecting the balance of the Th17/Treg axis and increasing the proportion of Th17 cells (160). Since IL-17 cannot only change the pathogenicity of periodontal microbiota but also lead to increased inflammation and more periodontal bone loss, the increase in the Th17/Treg ratio further aggravates the deterioration of diabetes periodontitis (71, 72).

#### 3.3.3 Host response modulation therapy

Host response modulation therapy aims to restore the balance between proinflammatory factors and anti-inflammatory mediators, prevent the development of periodontitis, and repair periodontal tissue destruction (179, 180).

The main kind of substances used in host response modulation therapy include nonsteroidal anti-inflammatory drugs (NSAIDs) (181), anti-cytokine therapy (182), sub-antimicrobial doses of doxycycline (SDD) (183), and specialized proresolving mediators (SPM) such as resolvins (184), maresins (185), lipoxins (186), probiotics (159), and other substances such as resveratrol (187) melatonin (188), and curcumin (189). Omega-3 polyunsaturated fatty acids (ω-3 PUFA) can change the neutrophil function, reduce inflammatory reactions, and improve host antioxidant capacity (190). They are represented by docosahexaenoic acid (DHA), which is the origin of marines and protectins, and eicosapentaenoic acid (EPA), which is related to the resolvin E series (RvE) (191).

NSAIDs are not an efficient modulation therapy (192). Systematic analysis also failed to prove that SDD treatment can bring significant improvement to diabetes patients with periodontitis (193). The use of ω-3 PUFAs can prevent excessive inflammatory processes by producing specialized proresolving mediators with effects on glycemic control and lipid profile (194). Some independent studies also suggested using resveratrol, curcumin, or melatonin as potential inflammatory immunomodulators for patients with diabetes periodontitis (195–197). However, current data are insufficient to demonstrate its absolute benefits for treating disease (192).

### 3.4 Oxidative stress

Oxidative stress (OS) is defined as “an imbalance between oxidants and antioxidants in favor of the oxidants, leading to a disruption of redox signaling and control and/or molecular damage,” and is closely related to the pathogenesis of chronic metabolic diseases such as diabetes mellitus and chronic inflammatory diseases such as periodontal disease (198, 199). Disorders in the redox balance and the resulting cellular dysfunction make a prooxidant environment that produces more ROS, while the body’s ability to scavenge free radicals decreases. Defense systems include antioxidant systems such as catalase (CAT), glutathione peroxidase (GPx), and Cu–Zn superoxide dismutase (SOD) and vitamins like vitamins E, A, and C (198, 200, 201).

OS is a common factor that causes the occurrence and development of diabetes and periodontitis through an upregulated host immune/inflammatory response and causes damage to vital biomolecules such as proteins, lipids, and DNA (198, 201, 202). Because the immune defense of diabetes patients is damaged, it is difficult to respond to the high level of subgingival pathogenic microorganisms in periodontitis patients, which will increase the destruction of periodontal tissue (203). At the same time, prooxidant states in periodontal tissue can lead to decreased insulin sensitivity, insulin resistance, and significant systemic effects (204). The coexistence of diabetes and periodontitis may have a synergistic effect, leading to more unbalanced redox control (204).

#### 3.4.1 Oxidative stress in diabetes mellitus

The production of ROS and the resulting oxidative stress state play a crucial role in the pathogenesis of diabetes, which is closely related to impaired glucose utilization, insufficient insulin secretion, and insulin resistance and ultimately leads to chronic hyperglycemia (200). It has been proven that the concentration of products of oxidative stress related to lipid peroxidation products, such as malondialdehyde (MDA), protein oxidation products (nitrotyrosine and carbonyl levels), and DNA oxidation markers such as 8-hydroxy-2′-deoxyguanosine (8-OHdG), in diabetes mellitus are a significant improvement, along with reduced antioxidant enzymatic (such as CAT, SOD and GPx) and nonenzymatic antioxidants (such as vitamins C and E) activity (198, 200, 205, 206).

Overproduction of ROS can occur through mechanisms associated with several pathways, such as polyol pathways, hexosamine pathways, PKC pathways, and AGE/RAGE pathways. Overactivation of all of the above signaling pathways ultimately leads to increased intracellular oxidative stress (207–209). The excessive production of ROS induced by hyperglycemia is conducive to inducing the polarization of M1-type proinflammatory macrophages, and activated polarized M1 macrophages are recruited to the inflammatory site where neutrophil-mediated respiratory burst occurs, where more proinflammatory mediators are released, which will produce more ROS and exacerbate the severity of oxidative stress (210). The bigger the OS that can cause damage to cellular macromolecules becomes, the greater the intensity of inflammatory reactions and mitochondrial dysfunction, ultimately leading to less insulin secretion and more insulin resistance (207, 209).

Studies have shown that excessive ROS can be produced through both nonmitochondrial pathways and mitochondrial inner membrane. Mitochondrial dysfunction may lead to intracellular calcium homeostasis disorders and ultimately reduce insulin sensitivity reduction in T2DM patients (211). ROS leads to the inhibition of antioxidant enzymes and the reduction of nonenzymatic antioxidants in systemic tissues, leading to the enhancement of chronic OS and aggravating the systemic symptoms of diabetes (212). Therefore, the excessive production of ROS induced by hyperglycemia, in addition to diluting the body’s antioxidant response, can also trigger a vicious cycle including hyperglycemia, oxidative stress, metabolic damage, and reduced insulin secretion or insulin resistance.

#### 3.4.2 Oxidative stress in periodontitis

Multitudinous experimental studies have shown there is a close relationship between periodontitis and OS (198, 213, 214). An increasing number of reports also have also shown that increased oxidative stress markers and total oxidation levels have been detected in the blood, saliva, and gingival crevicular fluid of patients with periodontitis, further confirming the link between periodontal tissue inflammation and OS (214, 215). Periodontitis can induce a sustained low-level inflammation of periodontal tissue, thereby increasing the overproduction of ROS and creating an environment with reduced antioxidant capacity. Nonsurgical periodontal treatment can reduce the level of OS markers in periodontal tissue (216).

Periodontal microbial pathogens activate the host immune response, leading to the recruitment of leukocytes, predominantly polymorphonuclear neutrophils and macrophages, from the bloodstream to the site of periodontal infection (217). The neutrophils bind to periodontal pathogenic bacteria by cell surface pattern recognition receptors such as TLR2 and TLR4, and subsequently, periodontal pathogens are eliminated through functional activity change of neutrophils such as phagocytosis and bactericidal and the formation of NETs. This process involves a powerful intracellular killing and respiratory burst of neutrophils, with excessive release of ROS, leading to subsequent microbial killing. ROS and its induced excessive activation of oxidative stress can have a destructive effect on host cells and are therefore considered a double-edged sword (217–220).

Consistent with the effect of OS on diabetes, OS in periodontal tissue can also promote the release of proinflammatory factors and activate the NF-κB signal pathway (221, 222). ROS exerts antimicrobial activities by exacerbating nucleic acid damage, protein misfolding, lipid peroxidation, and endoplasmic reticulum stress, or mitochondria stress, thereby accelerating autophagy and apoptosis of defective cells (223). Moreover, ROS can also play an indirect role in the destruction of alveolar bone caused by periodontitis and function as signaling molecules intercellularly or intracellularly in the osteoclastogenesis of periodontal ligament stem cells. Therefore, the excessive production of ROS can trigger the inflammatory mechanism and osteoclast formation, leading to periodontal inflammation and alveolar bone loss during the progression of periodontitis (224, 225).

#### 3.4.3 Diabetes mellitus increased periodontitis susceptibility via oxidative stress

In addition to the inflammatory reaction mediated by periodontal pathogens and the subsequent production of excessive ROS caused by neutrophils, several pieces of evidence show that hyperglycemia caused by diabetes can also lead to the accumulation of oxidative stress products in periodontal tissue. High glycemic levels induce ROS by overactivating polyol pathways, hexosamine pathways, PKC pathways, and AGE/RAGE pathways, increasing AGE formation, mitochondrial dysfunction, and a significant OS increment (226–228). The preexisting periodontal disease together with these pathologic mechanisms in diabetes, with increased OS and aggravated release of proinflammatory mediators, may be the reason why diabetes patients have more periodontal damage, and it also partly explains the current situation that diabetes patients are more susceptible to periodontitis.

As one of the main disease mechanisms, AGEs are a major link between diabetes and its complications. Research shows that in chronic periodontitis associated with diabetes, the accumulated AGEs in the periodontium increase (229). The synergistic effect of ROS and AGE/RAGE axes can lead to excessive periodontal damage related to diabetes (230). In addition, in the presence of AGEs, ROS can enhance cell autophagy by activating the ERK pathway and change the oxygen diffusion state by altering the permeability and structure of cell membrane, resulting in increased OS in periodontal tissue (231). Another study has proved that under the condition of diabetes, AGEs accumulated in periodontal tissue induce OS by inhibiting the Sirt1/Nrf2/HO-1 signal axis through RAGE and further promoting the production of IL-6 and IL-8 (232). AGEs in synergy with TNF-α can promote stronger OS in hPDLSCs *in vitro* and exhibit greater damage to periodontal tissue (233). AGEs can also reduce osteogenic differentiation of PDLSCs through OS (234). Another study shows that the excessive accumulation of ROS in periodontal tissue caused by diabetes will produce an extended oxidative environment, leading to the telomere damage of PDLSCs and ultimately damaging the mechanism of periodontal tissue repair and regeneration (235).

The products of OS and markers of the antioxidant system have been used to research the pathogenesis of diabetes and periodontitis and the interaction between them, but no consistent conclusion has been reached until now. Studies have shown an increase in total antioxidant capacity in periodontitis patients with T2DM (236). Along the same lines, the level of malondialdehyde in the periodontal tissue of T2DM patients was also significantly increased (237). On the contrary, another study concluded that the OS markers (8-OHdG) and periodontal clinical parameters of the patients affected by T2DM and periodontitis had a significant reduction (238). A case-control study showed that the activity of SOD decreased in patients with periodontal disease alone and increased in periodontitis patients with T2DM (239); however, the level of GPx was upregulated due to periodontitis and independent of the individual’s diabetes status (240), suggesting that diabetes increases gingival activity as an adaptive mechanism.

#### 3.4.4 Antioxidants

An effective approach to improving the defense against OS is to supplement antioxidants, including vitamins C and E and carotenoids (241), lycopene (242), α- and γ-tocopherol, β-cryptoxanthin, *N*-acetylcysteine (NAC) (243), polyphenolic compounds (244), such as flavonoids (245), zeaxanthin, and lutein. It has been reported that NAC alone has limited antioxidant effects, but when used together with glycine, GlyNAC can synergistically improve mitochondrial dysfunction and insulin secretion reduction, increase GPx synthesis, and reduce OS (241, 243).

Nrf2 is an inflammatory-related transcription factor that is crucial for balancing oxidative stress damage and the regulation of antioxidant responses. So the targeted treatment of the Nrf2/HO-1 axis can improve the periodontal injury caused by oxidative stress reaction and the subsequent amplified inflammatory response in diabetes patients with periodontitis (246, 247). Magnolol is derived from *Magnolia officinalis* and is beneficial for the improvement of diabetic complications. It has been shown to have antioxidant and anti-inflammatory properties and have a protective effect against periodontitis by activating the Nrf-2/HO-1 signaling axis (248, 249).

However, there is no sufficient evidence to prove that the use of antioxidants alone can produce perfect treatment results, but the use of antioxidants can improve the prognosis of diabetes periodontitis and help periodontal tissue elevate its response to periodontal basic treatment (250).

### 3.5 Alveolar bone resorption damage

Compared with periodontitis alone, periodontitis with diabetes causes more serious damage to periodontal tissue, especially the alveolar bone. The potential pathogenesis of increased periodontal tissue destruction in diabetes patients includes decreased collagen production, increased collagen decomposition activity, RANKL-mediated increased osteoclastogenesis, and diminished bone regeneration (30). Alveolar bone resorption is mainly mediated by RANKL through its receptor RANK, and osteoprotegerin (OPG) is an antagonist of RANKL, which can inhibit the generation of osteoclasts and play an important role in bone protection. The ratio of RANKL/OPG determines their impact on bone metabolism. The RANKL/OPG pathway plays a major role in diabetic periodontal inflammation and periodontal tissue destruction (251).

Santos et al. (252) found that the RANKL level and RANKL/OPG ratio in the gingival crevicular fluid of periodontitis patients with poorly controlled diabetes were higher than those of periodontitis patients with well-controlled diabetes. Another study showed that oral infection stimulated RANKL expression in osteocytes, causing obvious bone loss and increasing the functional activity of osteoclast, which were further enhancements by diabetes mellitus, but there was no bone loss or increment of osteoclastogenesis detected in diabetic transgenic mice in RANKL-deletion osteocytes (253). In periodontitis rats with T1DM, TNF-α could induce increased expression of sclerotin and RANKL in alveolar osteocytes, leading to further alveolar bone resorption and bone loss (254). Another study showed that hyperglycemia caused by diabetes could damage the function of PDLSCs, affect the ability of osteogenic differentiation, and have an adverse effect on the regeneration of alveolar bone (235).

### 3.6 Epigenetic changes

The host’s susceptibility to periodontitis depends not only on the influence of periodontal microorganisms but also on other factors such as genetic environmental factors, lifestyle, and so on. In recent years, it has been gradually noticed that diabetes affects the occurrence and development of periodontitis by changing the epigenetics of periodontal tissue (255).

A recent study shows that hyperglycemia caused by diabetes can cause 1,163 genes’ epigenetic changes in gingival tissue, accompanied by histological changes. The epigenetic and morphological changes of periodontal tissue may increase the susceptibility of periodontal disease in diabetes patients (19). Another study on people in South India shows that the polymorphism of the *RAGEG82S* gene may be a risk factor for periodontitis when diabetes is combined (256).

## 4 Conclusions and perspective

There is a positive association between diabetes mellitus and periodontitis; they promote the incidence of each other and are related to disease severity. Diabetes mellitus is a main risk factor for periodontitis and can increase the pathogenicity of the periodontal microbiota.

**Figure 1:**
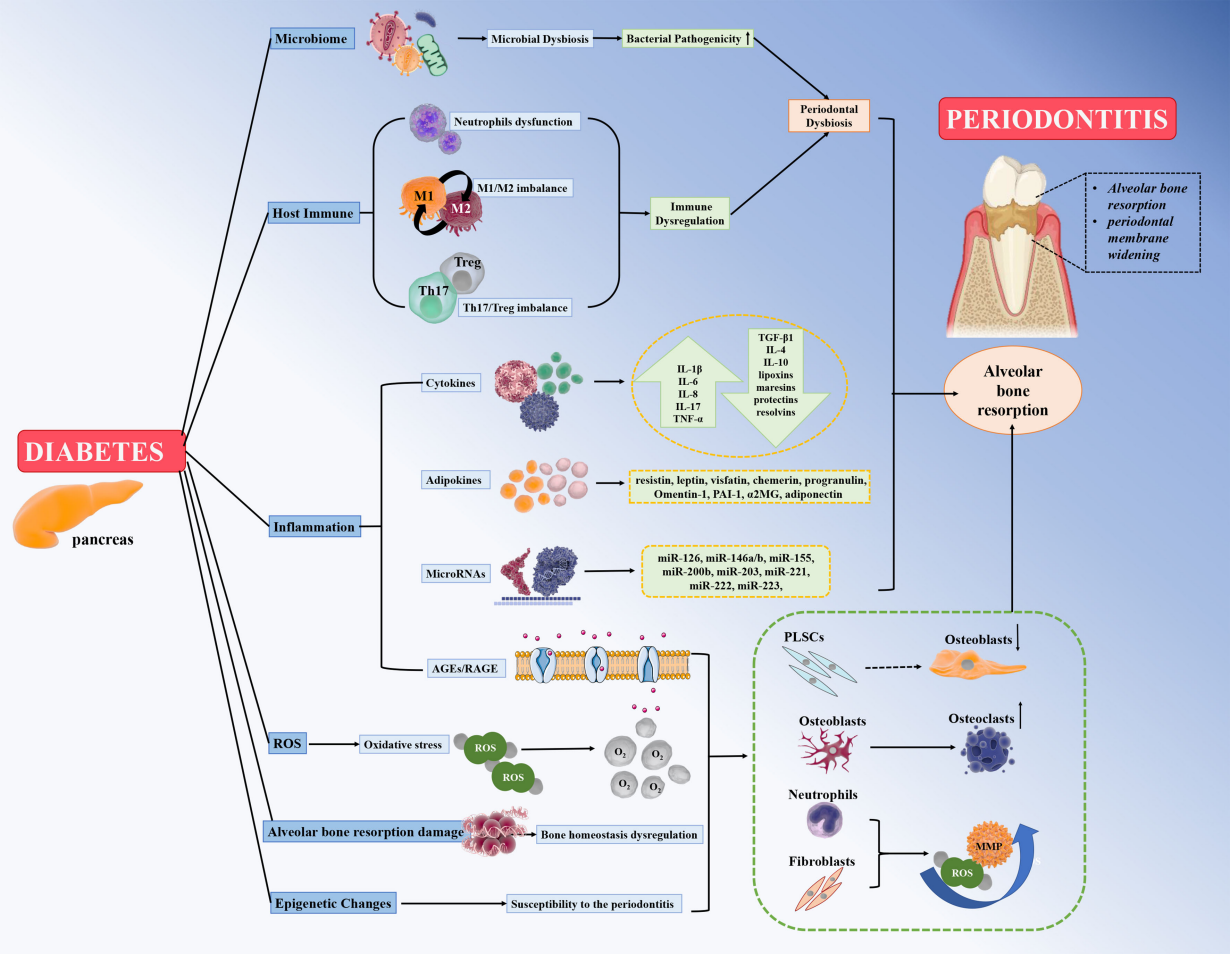
Diabetes mellitus can promote susceptibility to severe periodontitis by contributing to (1) microbiome factors; (2) host immune factors include neutrophil dysfunction, M1/M2 macrophage imbalance, and Th17/Treg imbalance; (3) enhanced inflammatory response to the bacterial challenge via cytokines, adipokines, miRNAs, and AGEs/RAGE; (4) oxidative stress and ROS; (5) alveolar bone resorption damage via RANKL/OPG ratio; and (6) epigenetic changes.

Animals with T2DM exhibit significant changes in the composition of the microbiota and a significant decrease in microbial diversity. At the same time, the shift from normal to dysbiotic microbiota in healthy individuals is greater than in diabetes patients, suggesting that diabetes mellitus can increase the inflammatory response to oral bacteria via cytokines, adipokines, AGE/RAGE, and miRNAs. Diabetes mellitus magnifies and strengthens the inflammatory response of bacterial attack in all aspects. The magnitude of the host immune response of periodontal cells in resisting the activity of the dysbiotic microbiota determines the host susceptibility and the more severe destruction of the periodontium. Moreover, hyperglycemia induces the production of ROS and enhances OS by AGEs/RAGEs, exacerbating periodontal destruction in diabetics. Furthermore, decreased collagen production, increased collagen decomposition activity, the RANKL/OPG ratio-mediated alveolar bone resorption damage, and the epigenetic changes in periodontal tissue induced by diabetes may also contribute to an increased susceptibility to periodontitis in diabetes mellitus patients. All the above biological processes that promote the susceptibility of diabetes mellitus to periodontitis are shown in **Figure 1**.

The reverse is also true; periodontitis is highly correlated with poor glycemic control in diabetes patients (10, 21–24). In addition, a prospective study showed that the occurrence of new diabetes was negatively related to the improvement of oral health (257). The mechanism of periodontitis affecting glycemic control in diabetes mellitus involves periodontal pathogens destroying the balance of intestinal microbiota, the spread of inflammatory mediators exacerbating systemic inflammation and metabolic damage, periodontal pathogens entering the blood causing bacteremia (30, 258–261), and periodontal pathogens reducing insulin production (262) or insulin resistance (263). Periodontal treatment can improve glycemic control of diabetes, but the evidence is still insufficient, and more clinical data are needed (25–29). New research showed that there was a direct relationship between the enhanced glycolysis of macrophages induced by hyperglycemia and training immunity (263), which may provide a theoretical basis for the two-way relationship between diabetes and periodontitis, and this new research field needs more exploration.

Furthermore, a better understanding of the mechanistic links between diabetes mellitus and periodontitis will benefit the identification of novel potential therapeutic targets, such as pro-resolution pathways, host response modulation therapy, Th17/Treg imbalance, antioxidant therapy, trained immunity, and gene modification. At the same time, there needs to be an interprofessional collaborative sense between endocrinologists and stomatologists while managing periodontitis patients with diabetes mellitus; endocrine physicians need to take care of periodontal diseases caused by diabetes and their effect on glycemic control; and patients with periodontitis should be counseled regarding their elevated risk of diabetes. In fact, studying the pathogenesis of the intersection network of diabetes mellitus and periodontitis can better explain the cross-interference of metabolic diseases and inflammatory diseases and provide a theoretical basis for new systemic holistic treatments.

## Author contributions

MZ: consulted, analyzed, and summarized the literature and drafted the manuscript. YX: consulted, analyzed, and summarized the literature. WG and QY: consulted the literature. CL: revised the manuscript. YL: designed the study and drafted and revised the manuscript. All authors contributed to the article and approved the submitted version.
